# Lower diastolic wall strain is associated with coronary revascularization in patients with stable angina

**DOI:** 10.1186/s12872-017-0739-3

**Published:** 2017-12-28

**Authors:** Jaehuk Choi, Min-Kyung Kang, Chaehoon Han, Sang Muk Hwang, Sung Gu Jung, Han-Kyul Kim, Kwang Jin Chun, Seonghoon Choi, Jung Rae Cho, Namho Lee

**Affiliations:** 1 Division of Cardiology, Hangang Sacred Heart Hospital, Hallym University Medical Center, Seoul, South Korea; 2 Cardiology Division, Kangnam Sacred Heart Hospital, Hallym University Medical Center, Seoul, South Korea; 3 Division of Cardiology, Department of Medicine, College of Medicine, Kangwon National University, Chuncheon, South Korea

**Keywords:** Echocardiography, Diastolic wall strain, Coronary revascularization

## Abstract

**Background:**

Left ventricular (LV) diastolic dysfunction occurs earlier in the ischemic cascade than LV systolic dysfunction and electrocardiographic changes. Diastolic wall strain (DWS) has been proposed as a marker of LV diastolic stiffness. Therefore, the objectives of this study were to define the relationship between DWS and coronary revascularization and to evaluate other echocardiographic parameters in patients with stable angina who were undergoing coronary angiography (CAG).

**Methods:**

Four hundred forty patients [mean age: 61 ± 10; 249 (57%) men] undergoing CAG and with normal left ventricular systolic function without regional wall motion abnormalities were enrolled. Among them, 128 (29%) patients underwent revascularization (percutaneous intervention: 117, bypass surgery: 11). All patients underwent echocardiography before CAG and the DWS was defined using posterior wall thickness (PWT) measurements from standard echocardiographic images [DWS = PWT(systole)-PWT(diastole)/PWT(systole)].

**Results:**

Patients who underwent revascularization had a significantly lower DWS than those who did not (0.26 ± 0.08 vs. 0.38 ± 0.09, *p* < 0.001). Age was comparable between the two groups (61 ± 9 vs. 60 ± 11, *p* = 0.337), but the proportion of males was significantly higher among patients who underwent revascularization (69 vs. 52%, *p* = 0.001). The LV ejection fraction was similar but slightly decreased (60.9 ± 5.7 vs. 62.4 ± 6.2%, *p* = 0.019) and the E/E’ ratio was elevated (10.3 ± 4.0 vs. 9.0 ± 3.1, *p* < 0.001) among patients who underwent revascularization. In multiple regression analysis, lower DWS was an independent predictor of revascularization (cut-off value: 0.34; sensitivity: 89%; AUC: 0.870; SE: 0.025; *p* < 0.001).

**Conclusion:**

DWS, a simple parameter that can be calculated from routine 2D echocardiography, is inversely associated with the presence of coronary artery disease and the need for revascularization.

## Background

Conditions causing chest pain or discomfort, such as an acute coronary syndrome or angina, have a potentially poor prognosis, emphasizing the importance of a prompt and accurate diagnosis [1]. For patients with acute coronary syndrome (ACS), 12-lead-electrocardiography (ECG), cardiac biomarkers, and echocardiography are used to confirm the diagnosis in combination with the characteristics of chest pain and conventional risk factors [1, 2]. Echocardiography is a good imaging modality for detecting a new loss of viable myocardium or new regional wall motion abnormality (RWMA) [1]. However, stable angina cannot be diagnosed or excluded by clinical assessment alone, and non-invasive functional imaging for myocardial ischemia, coronary computed tomography (CT) calcium scanning or angiography, and coronary angiography (CAG) are often needed. Non-invasive functional imaging for myocardial ischemia uses myocardial perfusion scintigraphy with single-photon emission computed tomography (MPS with SPECT), stress echocardiography, first-pass contrast-enhanced magnetic resonance (MR) perfusion or MR imaging for stress-induced wall motion abnormalities [3]. Using research by Genders et al. [4], the ESC 2013 guideline contains a similar table of estimated risk percentages to the NICE 2010 guideline. Those with an estimated risk of <15% should be presumed to not have coronary artery disease (CAD). If the estimated risk is >15%, the patient’s left ventricular ejection fraction (LV EF) is a key determinant factor. Therefore, cardiac imaging plays a pivotal role in this type of decision- decision-making through determination of the LV systolic function and subsequent selection of a relevant intervention. However, EF cannot be used as an independent parameter to estimate the probability of the presence of CAD, but it can be used to help choose further studies to detect CAD in combination with clinical assessments. Therefore, routine 2-D echocardiography is less useful in patients with stable angina than in patients with ACS.

An echocardiographic study published in 1972 showed abnormal motion of the LV posterior wall during angina pectoris [5]. Recently, a non-invasive, load-independent, and reproducible estimator of LV stiffness using 2-dimensional (2D) echocardiography, namely, diastolic wall strain (DWS), has been used to assess wall distensibility in the absence of LV systolic dysfunction [6]. Decreased DWS is associated with poor prognosis in patients with heart failure with preserved LV ejection fraction (HFpEF) [7] as well as in those with HF with reduced LVEF (HFrEF) [8] and is associated with adverse LV remodelling even in patients with normal LV systolic and diastolic function [9]. Therefore, the aims of this study were to evaluate the relationship between DWS and coronary revascularization as well as to evaluate echocardiographic and other parameters in patients with stable angina who underwent CAG.

## Methods

### Study design and participants

Of 2375 patients who underwent CAG from January 2013 to December 2015, 440 patients (57% male, average age = 60 years) who were diagnosed with or suspected to have stable angina were studied retrospectively. Patients were referred for evaluation of chest pain or dyspnoea by their general practitioner or by themselves. The character of the chest pain (typical, atypical, or nonanginal) was evaluated, and we excluded any patient with resting pain or suspected unstable angina. A variety of risk factors were recorded during the clinical assessment. Patients who had a prior history of CAD were excluded from this study regardless of their history of coronary stenting. All patients underwent CAG and pre-procedural EF by transthoracic echocardiography (TTE) and were sorted into two groups – the revascularization group (*n* = 128) and no revascularization group (*n* = 312). Patients with ACS, chronic kidney diseases, LV EF < 50%, regional wall motion abnormalities, arrhythmia, severe valvular heart diseases, pericardial diseases, thyroid disease, moderate to severe pulmonary hypertension, sepsis, or haemodynamic instability were excluded from this study. We also collected participant data regarding demographic, anthropometric, and laboratory parameters including cardiac biomarkers.

### Echocardiography

TTE was performed using standard techniques with a 2.5-MHz transducer. Standard 2-D and Doppler echocardiography was performed using a commercially available echocardiographic machine (Vivid 7R GE Medical System, Horten, Norway) with the same setup interfaced and a 2.5-MHz phased-array probe. With the study participants in the partial left decubitus position and breathing normally, the observer obtained images from the parasternal long and short axes as well as from the apical four chamber and two-chamber and long-axis views. The depth setting was optimized to display the LV on the screen as large as possible and the same field depth was kept for both the four and two-chamber apical views. The sector width was reduced to increase the spatial and temporal resolution. The LV end-diastolic dimensions (LV EDD), end-diastolic interventricular septal thickness (IVSTd), and end-diastolic LV posterior wall thickness (PWTd) were measured at end-diastole according to the standards established by the American Society of Echocardiography [10]. LV EF was determined by the biplane Simpson’s method. The maximal left atrial (LA) volume was calculated using the Simpson method and indexed to the body surface area. The LV mass was calculated using the Devereux formula: LV mass = 1.04[(LVEDD + IVSTd + PWTd)^3^ − (LVEDD)^3^] − 13.6. Thereafter, the LV mass index (LVMI) was calculated and indexed to the body surface area. DWS was calculated as [(PWTs) - (PWTd)/(PWTs)] using M-mode echocardiography [6, 7].

The mitral flow velocities were recorded in the apical four-chamber view. The mitral inflow measurements included the peak early (E) and peak late flow velocities (A) as well as the E/A ratio. The tissue Doppler of the mitral annulus movement was also obtained from the apical four-chamber view. A 1.5-mm sample volume was placed sequentially at the septal annular sites. An analysis was performed for the early diastolic (E’) and late diastolic (A’) peak tissue velocities. As a noninvasive parameter for LV stiffness, the LV filling index (E/E’) was calculated by the ratio of the transmitral flow velocity to the annular velocity. Adequate mitral and tissue Doppler image (TDI) signals were recorded in all patients.

The mean longitudinal global strain (GS) of LV was calculated from the apical 4,3,2-chamber views by speckle-tracking 2D–strain imaging [11].

### Carotid ultrasound

A high-resolution B-mode ultrasound (Vivid 7R GE Medical Systems, Horten, Norway) equipped with a 7.5-MHz linear array transducer was used for carotid ultrasonography. In the longitudinal view, the carotid intima-media thickness (IMT) was determined as the distance from the media adventitia interface to the intima lumen interface on the far wall in a region free of plaque [12]. The examiner assessed the presence of carotid plaques, which were defined as focal structures that encroached into the lumen by at least 100% of the surrounding IMT value. The common carotid artery IMT (CCA-IMT) was measured between the origin of the carotid bulb and a point 10 mm proximal to the CCA, and the carotid bulb IMT (CB-IMT) was measured in the carotid bulb region. The CCA-IMT and CB-IMT values were determined as the average of the maximum IMT of the left and right CCA and CB.

### Statistical analysis

All of the continuous data are expressed as the mean ± SD, and all of the categorical data are presented as percentages or absolute numbers. Continuous variables were analysed using Student’s t-test, and dichotomous variables were analysed using the chi-square test. In addition, multivariate analysis (logistic regression, SPSS for Macintosh, version 10.0.7a, SPSS, Inc., Chicago, Ill., USA) was performed. All variables that had a *p* value of 0.05 or less were considered statistically significant. The performance of continuous tests was assessed by receiver-operating-characteristic (ROC) curve.

## Results

### Clinical parameters of the study population

The clinical characteristics of the patients are shown in Table 1. The study population included 440 patients who underwent CAG (mean age 60 ± 10 years) and 128 patients who underwent coronary revascularization. Patients in the revascularization group were predominantly male (69% vs. 52%), and diabetes (27% vs. 17%) and current smoker status (31% vs. 22%) were more common in these patients. The incidence of coronary revascularization in men was 35% (87/248) and in women was 21% (40/192), which was statistically significantly higher in males (*p* = 0.001).

**Table 1:** Clinical parameters of the study population

	Revascularization (*n* = 128)	No (*n* = 312)	*p*
Age (years)	61.1 ± 9.4	60.0 ± 10.5	0.337
Male gender	88 (69%)	161 (52%)	0.001
Systolic blood pressure (mmHg)	123.6 ± 14.2	121.1 ± 15.4	0.126
Diastolic blood pressure	74.4 ± 9.5	73.5 ± 9.9	0.385
Heart rate (bpm)	66.7 ± 11.2	66.4 ± 12.4	0.809
Body mass index (kg/m^2^)	24.8 ± 3.4	25.2 ± 3.2	0.226
Hypertension	69 (54%)	140 (45%)	0.093
Diabetes	34 (27%)	54 (17%)	0.035
Stroke	5 (4%)	16 (5%)	0.806
Current smoker	40 (31%)	68 (22%)	0.051
Medications			
RASB	38 (30%)	82 (27%)	0.410
CCB	31 (25%)	74 (24%)	0.902
Statin	40 (32%)	84 (27%)	0.350
Aspirin	36 (29%)	67 (21%)	0.135
Clopidogrel	10 (7%)	14 (5%)	0.167
Diuretics	9 (7%)	28 (9%)	0.704
Beta-blocker	17 (14%)	32 (10%)	0.402
Trimetazidine	14 (11%)	19 (6%)	0.075

Data are mean ± standard deviation (SD) or or n (%)

*CHD* coronary heart disease, *RASB* Renin-Angiotensin system blocker, *CCB* calcium-channel blocker

### Echocardiographic parameters of the study population

**Fig. 1:**
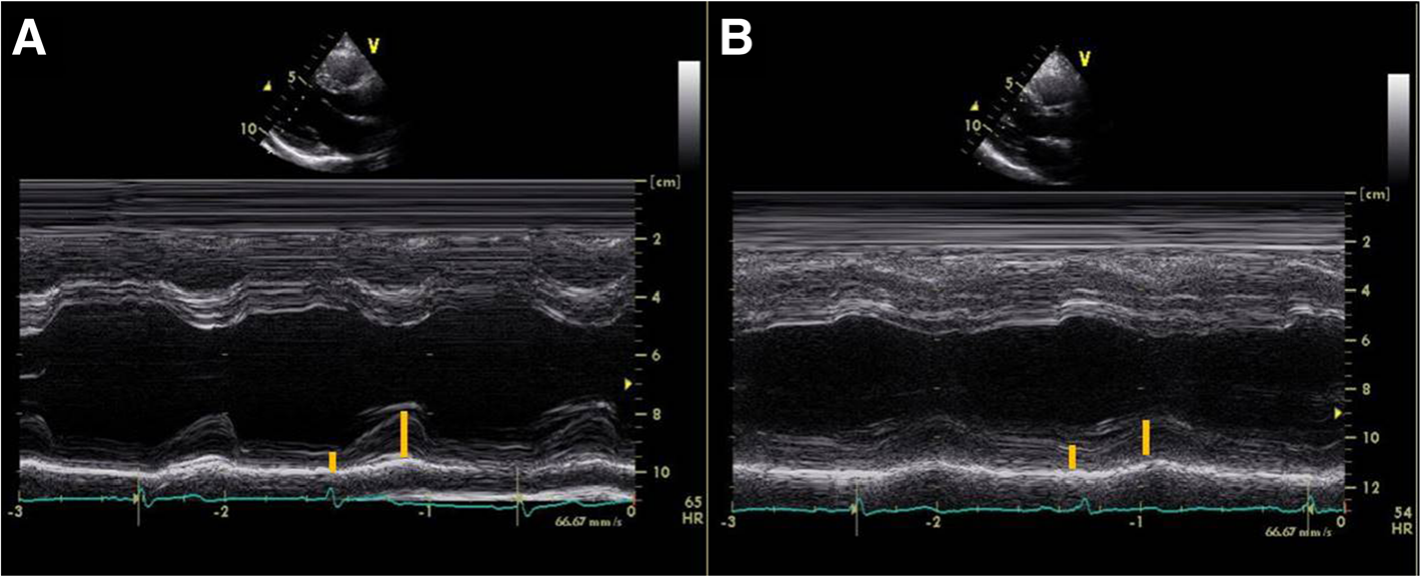
Echocardiographic images of M-mode from parasternal long-axis view, a patient with normal diastolic wall strain (DWS, **a**) and a patient with decreased DWS (**b**)

One hundred twenty-eight patients underwent coronary revascularization (percutaneous intervention: 113; bypass surgery: 14) among the 440 patients who underwent CAG. Echocardiographic measurements of the study population are shown in Table 2. The entire study population had a normal cardiac size and systolic function, but patients in the revascularization group had a slightly but significantly decreased LV systolic end-dimension, EF, and GS. Patients in the revascularization group also had a lower LV posterior wall thickness during systole and DWS. Figure 1 shows normal (a) vs. decreased DWS (b). Patients who underwent revascularization had a more advanced diastolic dysfunction and poorer diastolic parameters (lower E’ velocity and elevated E/E’). In the analysis according to gender, ± 0.09 vs. 8 ± 0.10, *p* = 0.001). However, the mean DWS was significantly lower in the coronary revascularization group in both men (0.38 ± 0.08 vs. 0.28 ± 0.07, *p* < 0.001) and women (0.42 ± 0.08 vs. 0.29 ± 0.10, *p* < 0.001).

**Table 2:** Echocardiographic parameters of the study population

	Revascularization (*n* = 128)	No (*n* = 312)	*p*
LAVI (ml/m^2^)	22.7 ± 6.9	22.9 ± 8.1	0.862
LVMI (g/m^2^)	94.6 ± 18.9	91.1 ± 22.2	0.119
LV SWTd (mm)	9.1 ± 1.4	8.9 ± 1.4	0.147
LV SWTs	13.4 ± 1.7	13.0 ± 1.8	0.033
LV PWTd	9.0 ± 1.3	8.7 ± 1.3	0.045
LV PWTs	12.6 ± 1.4	13.9 ± 1.6	<0.001
DWS	0.26 ± 0.08	0.38 ± 0.09	<0.001
LV EDD (mm)	49.9 ± 3.5	49.7 ± 4.1	0.632
LV ESD	32.4 ± 2.9	31.7 ± 3.6	0.049
LV EF (%)	60.9 ± 5.6	62.4 ± 6.2	0.019
GS (%)	−17.4 ± 2.5	−18.3 ± 2.9	0.016
E (cm/s)	63.6 ± 15.7	63.8 ± 15.6	0.901
A (cm/s)	74.6 ± 21.2	71.5 ± 17.8	0.122
E/A ratio	0.90 ± 0.31	0.93 ± 0.32	0.369
DT (ms)	208.6 ± 44.7	202.0 ± 44.2	0.162
E’ (cm/s)	6.7 ± 2.1	7.6 ± 2.5	0.001
A’ (cm/s)	9.3 ± 1.8	9.3 ± 1.8	0.970
E’/A’	0.75 ± 0.29	0.85 ± 0.34	0.005
E/E’	10.3 ± 4.0	9.1 ± 3.1	<0.001
S′ (cm/s)	7.4 ± 1.5	7.6 ± 1.6	0.221
Diastolic grade			<0.001
normal	22 (19%)	94 (31%)	
Grade 1	83 (70%)	201 (66%)	
Grade 2	14 (11%)	9 (3%)	

Data are represented as mean ± SD or n (%)

*LAVI* left atrial volume index, *LVMI* left ventricular mass index, *LVEDD and ESD* LV end-diastolic and systolic dimension, *EF* ejection fraction, *GS* global strain, *DT* deceleration time

### Laboratory findings and other parameters

Table 3 shows the laboratory parameters and results from the carotid ultrasound. Serum creatinine was higher in patients in the revascularization group. The mean IMT and maximal plaque thickness were higher in patients in the revascularization group.

**Table 3:** Laboratory and other parameters

	Revascularization (*n* = 128)	No (*n* = 312)	*p*
Creatinine (mg/dl)	0.99 ± 1.15	0.81 ± 0.56	0.029
Total cholesterol	167.4 ± 42.7	163.0 ± 36.7	0.301
LDL	100.0 ± 36.2	95.9 ± 32.3	0.278
HDL	42.2 ± 10.8	43.9 ± 11.9	0.187
TG	139.8 ± 74.6	125.2 ± 75.6	0.088
hsCRP	2.49 ± 3.67	2.04 ± 3.11	0.417
BNP	44.1 ± 90.6	32.4 ± 52.8	0.117
CK-MB	6.79 ± 43.42	1.74 ± 2.17	0.196
Troponin I	1.60 ± 10.80	0.11 ± 0.54	0.125
Mean IMT (mm)	0.77 ± 0.24	0.70 ± 0.16	0.009
Carotid plaque (mm)	80 (90%)	166 (81%)	0.125
Max. plaque (mm)	2.36 ± 0.70	2.02 ± 0.57	<0.001
ABI,rt	1.15 ± 0.12	1.17 ± 0.07	0.176
ABI,lt	1.13 ± 0.11	1.16 ± 0.08	0.103

Data are mean ± standard deviation (SD) or or n (%)

*CHD* coronary heart disease, *LAVI* left atrial volume index, *LV EDD/ESD* left ventricular end-diastolic and systolic dimension, *EF* ejection fraction, *LVMI* left ventricular mass index, *DT* deceleration time, *RVSP* estimated right ventricular systolic pressure

### The angiography results of 127 patients who underwent coronary revascularization

Table 4 shows the angiography results of 127 patients who underwent coronary revascularization. Fifty-six patients had single vessel disease (VD), and 71 patients had multi-vessel involvement. Most patients underwent one stent implantation, and one or more stent implantations were performed in 32 patients. The left anterior descending artery (LAD) was the most common target lesion, and 26% of patients had multiple lesions. All patients underwent coronary revascularization-confirmed complete revascularization (CR). CR was defined as a final angiography result without coronary stenosis ≥70% in major epicardial vessels or stenosis ≥50% in the left main [13].

**Table 4:** The angiography results of 127 patients who performed coronary revascularization

Characteristics	Number of patients (%)
1 Vessel disease (VD)	56 (44%)
2 VD	45 (35%)
3 VD	26 (21%)
Number of stents	
0^a^	19 (15%)
1	76 (60%)
2	26 (20%)
3	5 (4%)
4	1 (1%)
Target lesion	
Involvement of left main	9 (7%)
Left anterior descending artery	46 (36%)
Left circumflex artery	15 (12%)
Right coronary artery	24 (20%)
Multiple	33 (26%)
Proximal	50 (39%)
Mid to distal	44 (35%)
Both (tandem lesion)	33 (26%)

^a^Of the 19 patients who did not undergo stent implantation, 14 patients underwent bypass surgery, 4 patients underwent balloon angioplasty, and 1 patient underwent thrombus suctioning

### Univariate analysis

Univariate analysis showed that several clinical, echocardiographic, and other factors had predictive value for coronary revascularization. A lower DWS, decreased GS, diastolic dysfunction, elevated E’ velocity, male gender, diabetes, current smoking, higher IMT and higher maximal plaque thickness were associated with coronary revascularization in patients with clinical suspicion of stable angina in those who underwent CAG (Table 5).

**Table 5:** Uni-and multivariate analysis of the determinants of coronary revascularization in patients with stable angina

	Odds ratio	95% CI	*p*
Univariate analysis			
Diastolic wall strain	0.987	0.984–0.990	< 0.001
Left ventricular EF	0.996	0.958–1.037	0.856
Left ventricular global strain	0.888	0.805–0.979	0.017
E/E’	1.111	1.046–1.179	0.001
Diastolic grading	1.825	1.117–2.8983	0.016
Male gender	2.076	1.344–3.207	0.001
Diabetes	1.721	1.055–2.809	0.030
Current smoking	1.624	1.025–2.574	0.039
Creatinine	1.320	0.973–1.791	0.075
Mean carotid IMT	7.687	1.851–31.926	0.005
Maximal plaque	2.350	1.518–3.636	<0.001
Multivariate analysis			
Diastolic wall strain	0.875	0.806–0.950	0.001
Left ventricular global strain	1.061	0.913–1.232	0.440
E/E’	1.003	0.881–1.143	0.960
Diastolic grading	1.656	0.720–3.811	0.235
Male gender	1.211	0.512–2.864	0.663
Diabetes	0.668	0.281–1.584	0.359
Mean carotid IMT	13.041	1.277–124.827	0.025
Maximal plaque	1.779	0.985–3.215	0.073

*EF* ejection fraction, *IMT* intima-medial thickness

### Multivariate analyses

**Fig. 2:**
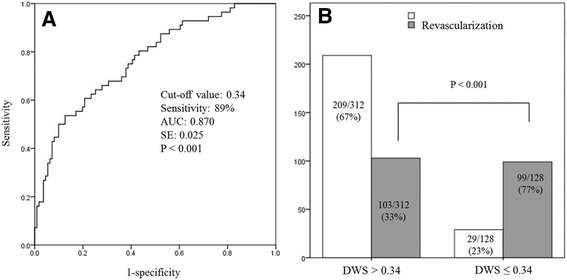
**a** ROC curve showing the predictive value of DWS (less than 0.34) in coronary revascularization (sensitivity of 89% and specificity of 48%, AUC: 0.870, SE: 0.025, *p* < 0.001, **a**). **b** Comparison of the proportion of patients who underwent coronary revascularization classified according to DWS (> 0.34 vs. 0.34 or less)

Among the variables found to be correlated with coronary revascularization, a lower DWS (OR: 0.920, CI: 0.862–0.981, *p* = 0.011) and higher IMT (OR: 12.629, CI: 1.277–124.817, *p* = 0.030) were found to be independently related to coronary revascularization (Table 5). To better analyse the predictive value of the relationship between DWS and coronary revascularization, we used ROC curve analysis to determine if any threshold value of DWS existed. Inspection of the ROC curve showed that DWS < 0.34 was associated with the need for coronary revascularization (sensitivity of 89% and specificity of 48%, AUC: 0.870, SE: 0.025, *p* < 0.001, Fig. 2a). Figure 2b presents the proportion of patients who underwent coronary revascularization classified according to DWS (> 0.34 vs. 0.34 or less). There was an absolute increase in the proportion of patients undergoing revascularization at a DWS value of 0.34 or lower.

## Discussion

The novel finding of this study is that decreased DWS was associated with coronary revascularization in patients with stable angina. The important clinical implication of this finding is that lower DWS may be helpful to predict the need for coronary revascularization in patients with normal pre-procedural LVEF. This suggests a possible role of echocardiography in the diagnosis of stable angina.

Several conditions of either traumatic or atraumatic aetiologies can cause chest pain. To diagnose or exclude ACS, which is a life-threatening condition, prompt evaluation of chest pain using an algorithmic approach is important [2]. The most common causes of chest pain in outpatients are musculoskeletal and gastrointestinal causes; 10% have stable angina, 5% have respiratory conditions, and approximately 2 to 4% have acute myocardial ischaemia [14]. The target population of this study was the 10% of patients with stable angina. Among patients with suspected stable angina according clinical, laboratory, and imaging studies, 29% (128/440) of patients needed coronary revascularization (percutaneous intervention: 113; bypass surgery: 14). However, stable angina could not be ruled out in all patients who did not undergo coronary revascularization. CAG was not an absolute indication in at least 312 study subjects. However, sometimes it is not feasible to perform non-invasive functional tests, such as the treadmill test or imaging tests (MPS with SPECT, stress echocardiography or MR perfusion) [3], in clinical practice. Hence, it is the standard practice at our centre to perform CAG if the suspicion of stable angina is high after clinical, laboratory and imaging studies (chest X-ray, ECG, or echocardiography). If pre-procedural echocardiography in patients with stable angina had some predictable parameters, it would be helpful to determine the patients in whom CAG is indicated. Retrospectively, 312 (71%) of our subjects did not require CAG. Non-invasive imaging plays a multifactorial role in patients with a known or suspected myocardial infarction. In particular, the advantages of echocardiography are to assess the cardiac structure and function and to detect RWMA (myocardial thickness, thickening and motion) [1]. Echocardiographic contrast agents can improve the visualization of the endocardial border and assess myocardial perfusion [15]. Tissue Doppler and strain imaging are very helpful tools for the quantification of global and regional function of LV [11, 15]. However, these modalities have been primarily used in the setting of myocardial infarction. Therefore, we investigated echocardiographic parameters other than LV function or RWMA in the setting of stable angina.

It follows from the concept of the “ischaemic cascade” [16] that exercise or pharmacologic stress tests (ECG, echocardiography, nuclear test, etc.) are the major tests for evaluating coronary artery stenosis with a high accuracy and prognostic value in patients with clinical suspicion of myocardial ischaemia [17–20]. However, performing these tests before deciding to perform CAG is not always possible in clinical practice due to limitations of time and cost or due to contraindications. Considering the ischaemic cascade (the onset of ischaemia is followed by LV diastolic dysfunction, LV systolic dysfunction, ECG changes and angina, in that order) [16], we hypothesized that the evaluation of LV diastolic dysfunction might be helpful in patients with suspected stable angina. Fogelman et al. compared multiple posterior wall echograms taken before, during, immediately after, and 5 min after exercise in normal subjects vs. patients with angina; the angina patients showed not only a significantly decreased resting diastolic posterior wall velocity but also a remarkable slowing of the diastolic posterior wall velocity during angina [5]. DWS, a non-invasive, load-independent, and reproducible estimator of LV stiffness using 2D echocardiography, is used to assess wall distensibility in the absence of LV systolic dysfunction [6]. Decreased DWS means increased diastolic myocardial stiffness and is associated with poor prognosis in patients with HFpEF [7] and HFrEF [8]. Hypertension can cause myocardial fibrosis in association with increased deposition of myocardial collagen, and myocardial fibrosis is responsible for myocardial stiffness. Even in patients with treated hypertension, decreased DWS (cut-off value <0.34) is associated with LV diastolic dysfunction [21]. The cut-off value of DWS for predicting coronary revascularization in our study was also 0.34 (sensitivity: 89%). Takagi et al. reported that DWS is inversely correlated with post-exercise E/E’ in elderly patients without obvious myocardial ischaemia, and DWS ≤ 0.33 was defined as low DWS [22]. In addition, low DWS (≤ 0.33) is associated with adverse LV remodelling (higher LV end-systolic volume and increased LV mass index) even in patients with normal LV diastolic function [9].

Atrial fibrillation (AF), the most common cardiac arrhythmia, is associated with the LV diastolic dysfunction [23]. Kang et al. observed that diastolic dysfunction (elevated E velocity, E/E’) and dys-synchronous LA were associated with the occurrence of stroke in patients with paroxysmal AF, even in those with a similar CHAD (except prior stroke) score [24]. Uetake et al. reported that patients with AF had lower DWS than controls (0.35 vs. 0.41) and that decreased DWS (<0.380) was a strong determinant of the prevalence of AF in patients with paroxysmal AF and structurally normal hearts [25].

Therefore, decreased DWS ≤ 0.33 or 0.34 is definitely associated with increased LV stiffness and diastolic dysfunction in various diseases [7–9, 21, 22, 25] and is associated with poor prognosis in patients with HFpEF, HFrEF [7, 8] and AF [25].

### Limitations

This study has a few limitations. First, few patients showed elevated pre-procedural cardiac biomarkers. Suspected ACS patients (typical chest pain, RWMA by echocardiography, ST elevation, or significant ST change in association with elevated cardiac biomarkers) were excluded from this study. Only a few patients who were very stable did not have resting chest pain and showed normal LVEF by echocardiography without discernible RWMA were included. Despite the insensitive and subjective nature of the echocardiographic evaluation of RWMA, the patients included in this study were not high-risk patients. Rarely, cardiac troponin can be elevated if the angina episode is prolonged in patients with chronic stable angina [26]. Second, we evaluated only epicardial coronary obstructions that needed revascularization. Therefore, microvascular angina or coronary spasm was not considered in this study. Third, we did not perform functional studies before CAG in all patients. Finally, the relatively small study population was a major limitation.

## Conclusions

In summary, decreased DWS is associated with coronary revascularization in patients with stable angina. The important clinical implication of this finding is that lower DWS may be helpful to predict coronary revascularization in patients with normal pre-procedural LV EF. This implies a possible role of echocardiography in the diagnosis of stable angina.

## Abbreviations

ACS: Acute coronary syndrome
CAD: Coronary artery disease
CAG: Coronary angiography
CB: Carotid bulb
CCA: Common carotid artery
CR: Complete revascularization
DWS: Diastolic wall strain
ECG: 12-lead-electrocardiography
EF: Ejection fraction
GS: Global strain
HFpEF: Heart failure with preserved LV ejection fraction
HFrEF: Heart failure with reduced LV ejection fraction
IMT: Intima-media thickness
IVSTd: End-diastolic interventricular septal thickness / PWTd: end-diastolic LV posterior wall thickness
LA: Left atrial
LV EDD: LV end-diastolic dimensions
LV: Left ventricular
LVMI: Left ventricular mass index
MPS with SPECT: Myocardial perfusion scintigraphy with single photon emission computed tomography
MR: Magnetic resonance
ROC: Receiver-operating-characteristic
RWMA: Regional wall motion abnormality
SD: Standard deviation
TDI: Tissue Doppler image
TTE: Transthoracic echocardiography
